# Antibody feedback contributes to facilitating the development of Omicron-reactive memory B cells in SARS-CoV-2 mRNA vaccinees

**DOI:** 10.1084/jem.20221786

**Published:** 2022-12-13

**Authors:** Takeshi Inoue, Ryo Shinnakasu, Chie Kawai, Hiromi Yamamoto, Shuhei Sakakibara, Chikako Ono, Yumi Itoh, Tommy Terooatea, Kazuo Yamashita, Toru Okamoto, Noritaka Hashii, Akiko Ishii-Watabe, Noah S. Butler, Yoshiharu Matsuura, Hisatake Matsumoto, Shinya Otsuka, Kei Hiraoka, Takanori Teshima, Masaaki Murakami, Tomohiro Kurosaki

**Affiliations:** 1 Laboratory of Lymphocyte Differentiation, WPI Immunology Frontier Research Center, Osaka University, Osaka, Japan; 2 Division of Medical Research Support, Advanced Research Support Center, Ehime University, Ehime, Japan; 3 Translational Research Center, Ehime University Hospital, Ehime, Japan; 4 Laboratory of Immune Regulation, WPI Immunology Frontier Research Center, Osaka University, Osaka, Japan; 5 Laboratory of Virus Control, Research Institute for Microbial Diseases, Osaka University, Osaka, Japan; 6 Laboratory of Virus Control, Center for Infectious Disease Education and Research, Osaka University, Osaka, Japan; 7 Institute for Advanced Co-Creation Studies, Research Institute for Microbial Diseases, Osaka University, Osaka, Japan; 8 KOTAI Biotechnologies, Inc., Osaka, Japan; 9 Division of Biological Chemistry and Biologicals, National Institute of Health Sciences, Kanagawa, Japan; 10 Department of Microbiology and Immunology, The University of Iowa, Iowa City, IA, USA; 11 Department of Traumatology and Acute Critical Medicine, Osaka University Graduate School of Medicine, Osaka, Japan; 12 Department of Surgery, National Hospital Organization Hakodate National Hospital, Hokkaido, Japan; 13 Division of Laboratory and Transfusion Medicine, Hokkaido University Hospital, Sapporo, Japan; 14 Department of Hematology, Faculty of Medicine, Hokkaido University, Sapporo, Japan; 15 Molecular Psychoimmunology, Institute for Genetic Medicine, Graduate School of Medicine, Hokkaido University, Sapporo, Japan; 16 Team of Quantum immunology, Institute for Quantum Life Science, National Institute for Quantum and Radiological Science and Technology, Chiba, Japan; 17 Division of Molecular Neuroimmunology, Department of Homeostatic Regulation, National Institute for Physiological Sciences, National Institutes of Natural Sciences, Aichi, Japan; 18 Center for Infectious Disease Education and Research, Osaka University, Osaka, Japan; 19 Laboratory for Lymphocyte Differentiation, RIKEN Center for Integrative Medical Sciences, Kanagawa, Japan

## Abstract

In contrast to a second dose of the SARS-CoV-2 mRNA vaccine, a third dose elicits potent neutralizing activity against the Omicron variant. To address the underlying mechanism for this differential antibody response, we examined spike receptor-binding domain (RBD)–specific memory B cells in vaccinated individuals. Frequency of Omicron-reactive memory B cells increased ∼9 mo after the second vaccine dose. These memory B cells show an altered distribution of epitopes from pre-second memory B cells, presumably due to an antibody feedback mechanism. This hypothesis was tested using mouse models, showing that an addition or a depletion of RBD-induced serum antibodies results in a concomitant increase or decrease, respectively, of Omicron-reactive germinal center (GC) and memory B cells. Our data suggest that pre-generated antibodies modulate the selection of GC and subsequent memory B cells after the second vaccine dose, accumulating more Omicron-reactive memory B cells over time, which contributes to the generation of Omicron-neutralizing antibodies elicited by the third vaccine dose.

## Teaser

Inoue et al. show that the antibody feedback plays a role in generating Omicron-reactive memory B cells in SARS-CoV-2 mRNA vaccinees.

## Introduction

Since the outbreak of COVID-19 in late 2019, several SARS-CoV-2 variants of concern have continuously emerged in the past years. Among them, the Omicron BA.1 (B.1.1.529) variant, harboring ∼15 mutations in the spike receptor-binding domain (RBD), showed a profound effect on evading the neutralizing antibody responses in those received two-dose of mRNA vaccination (Pfizer-BioNTech BNT162b2 or Moderna mRNA-1273). In fact, epidemiologic data suggested that weak or undetectable neutralizing antibodies against Omicron variant were induced in serum IgG after a two-dose of mRNA vaccination. In contrast, individuals boosted with a third dose of mRNA vaccine encoding the original Wuhan spike protein induced potent neutralizing serum activity against Omicron, and were highly protected from infection (Garcia-Beltran et al., 2022; Gruell et al., 2022; Muik et al., 2022; Schmidt et al., 2022).

In addition to the antibody induction, mRNA vaccination elicits the generation of SARS-CoV-2–specific memory B cells, which represent a second layer of immune protection through quick differentiation into antibody-secreting plasma cells upon re-encountering antigens. Indeed, a recall response from memory B cells was highlighted as a key factor for the protection from severe pathology in the lungs of nonhuman primates (Gagne et al., 2022). Furthermore, memory B cells can persist for a long period and evolve due to the progressive acquisition of somatic hypermutations (SHM) through germinal center (GC) reaction (Gaebler et al., 2021; Muecksch et al., 2022; Wang et al., 2021). In fact, SARS-CoV-2 mRNA vaccination induced a robust and persistent GC response in humans (Kim et al., 2022; Mudd et al., 2022; Turner et al., 2021). Hence, memory B cells are able to possess a diverse antibody repertoire, allowing for an adaptive response against the pathogen upon re-infection, particularly in the case of variant pathogen infections (Leach et al., 2019; Purtha et al., 2011; Wong et al., 2020).

In this study, to address the underlying mechanism of the differential neutralizing antibody responses against the Omicron variant between the second and the third vaccine dose, we analyzed memory B cells and their encoded antibodies in vaccinated individuals and used an immunized mouse model system for proof of concept. Our results suggest that acutely produced Omicron-non-cross-reactive antibodies help skew the memory B cells toward more Omicron-cross-reactivity during a two-dose immune response, thereby at least partly contributing to the generation of Omicron-neutralizing antibodies upon a third vaccine dose.

## Results

### Study design and cohorts

**Figure 1.**
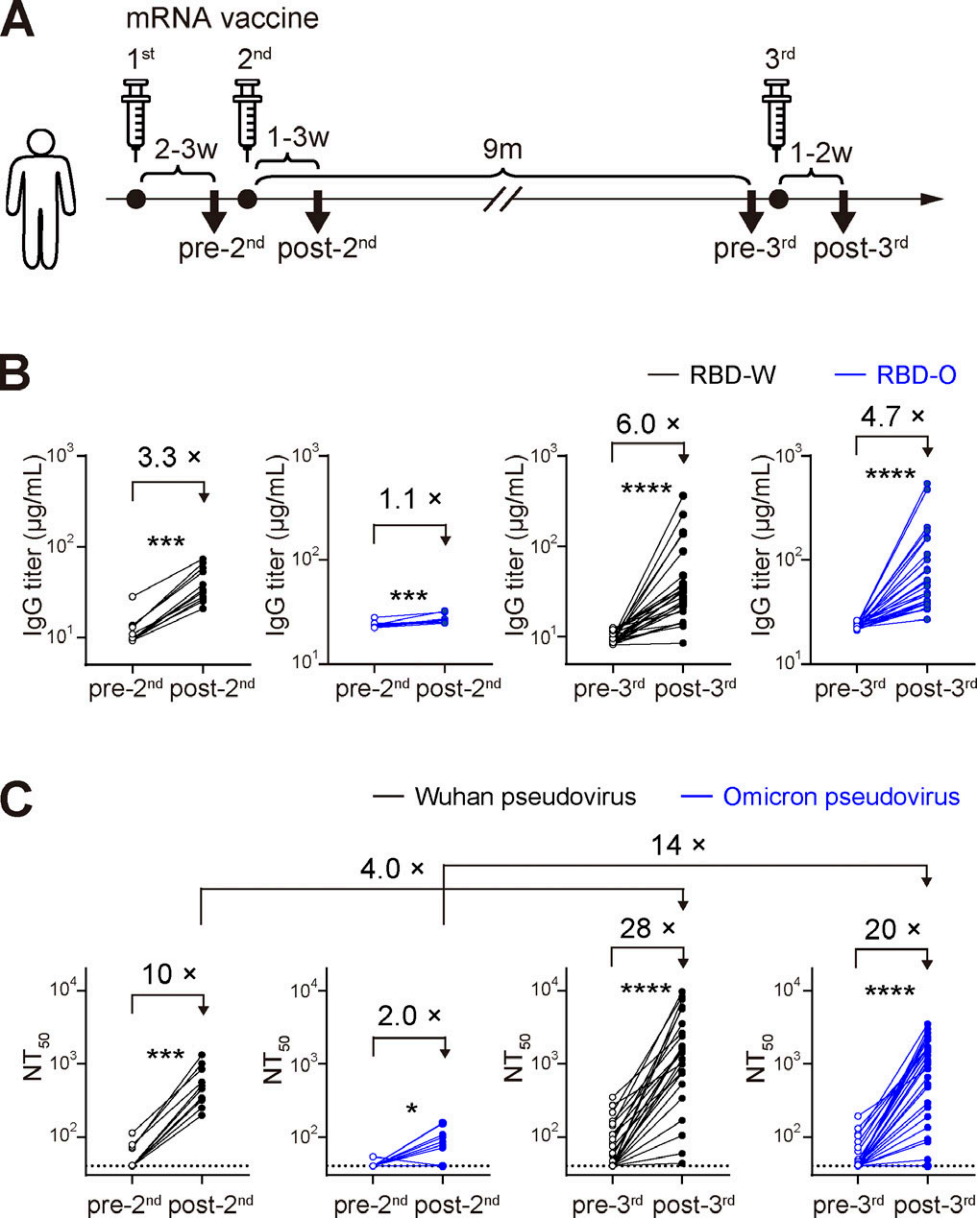
**Plasma antibody analysis from mRNA-vaccinated human cohorts. (A)** Schematic of sample collection. Plasma and PBMCs were collected from 2 to 3 wk after the first vaccination (pre-2nd, *n* = 12), 1–3 wk after the second vaccination (post-2nd, *n* = 12), ∼9 mo after the second vaccination (pre-3rd, *n* = 26), and 1–2 wk after the third vaccination (post-3rd, *n* = 26). **(B)** Plasma anti–RBD-W or RBD-O IgG titer quantified by ELISA and expressed as relative quantity of EY6A antibody. **(C)** Plasma neutralization activity against VSV pseudotyped with SARS-CoV-2 Wuhan or Omicron BA.1 spike protein expressed as NT_50_ values. **(B and C)** Experiments were performed in duplicate. Lines connect the longitudinal samples. *, P < 0.05; ***, P < 0.001; ****, P < 0.0001 by Wilcoxon test.

We examined the immune responses to three doses of the Pfizer-BioNTech (BNT162b2) or Moderna (mRNA-1273) mRNA vaccine in a longitudinal cohort of 35 volunteers with no prior history of COVID-19 diagnosis and no serological evidence of previous SARS-CoV-2 infection. Plasma and peripheral blood mononuclear cells (PBMCs) samples were collected at four different time points: 2–3 wk after the first vaccine dose (pre-2nd, *n* = 12), 1–3 wk after the second vaccine dose (post-2nd, *n* = 12), ∼9 mo after the second vaccine dose (pre-3rd, *n* = 26), and 1–2 wk after the third vaccine dose (post-3rd, *n* = 26; Fig. 1 A). Detailed cohort information is provided in Table S1.

### Antibody responses

Here, we focused on IgG responses to the SARS-CoV-2 spike RBD, as the RBD includes the major epitopes of neutralizing antibodies and IgG is a major contributor to the neutralizing activity of plasma antibodies compared to IgA (Andreano et al., 2021; Barnes et al., 2020; Cao et al., 2020; Kotaki et al., 2022; Piccoli et al., 2020; Rogers et al., 2020; Zost et al., 2020). An ELISA showed that plasma IgG anti-RBD binding activity against both Wuhan-Hu-1 and Omicron BA.1 RBDs (designated as RBD-W and RBD-O, respectively) were significantly increased after the third vaccine dose. By contrast, only a barely detectable increase of anti–RBD-O titer was observed after the second vaccine dose (Fig. 1 B). Plasma neutralizing activity was measured using a vesicular stomatitis virus (VSV) pseudotyped with Wuhan-Hu-1 or Omicron BA.1 spike protein (Fig. 1 C). The second and the third vaccine doses induced 10-fold and 28-fold increases, respectively, in the geometric mean half-maximal neutralizing titer (NT_50_) against the Wuhan pseudovirus. Of note, consistent with prior studies (Garcia-Beltran et al., 2022; Gruell et al., 2022; Muik et al., 2022; Schmidt et al., 2022), the third vaccine dose induced a potent neutralizing response against Omicron pseudovirus by 20-fold, in contrast to a limited increase after the second dose. Given that neutralizing titers toward Beta and Delta variants are also effectively boosted by the third vaccine dose (Muik et al., 2022), these results demonstrate that the antibody responses acquire a significant breadth by the third dose of mRNA vaccination with the original Wuhan spike protein.

### Flow cytometry analysis of memory B cells and plasmablasts

**Figure 2.**
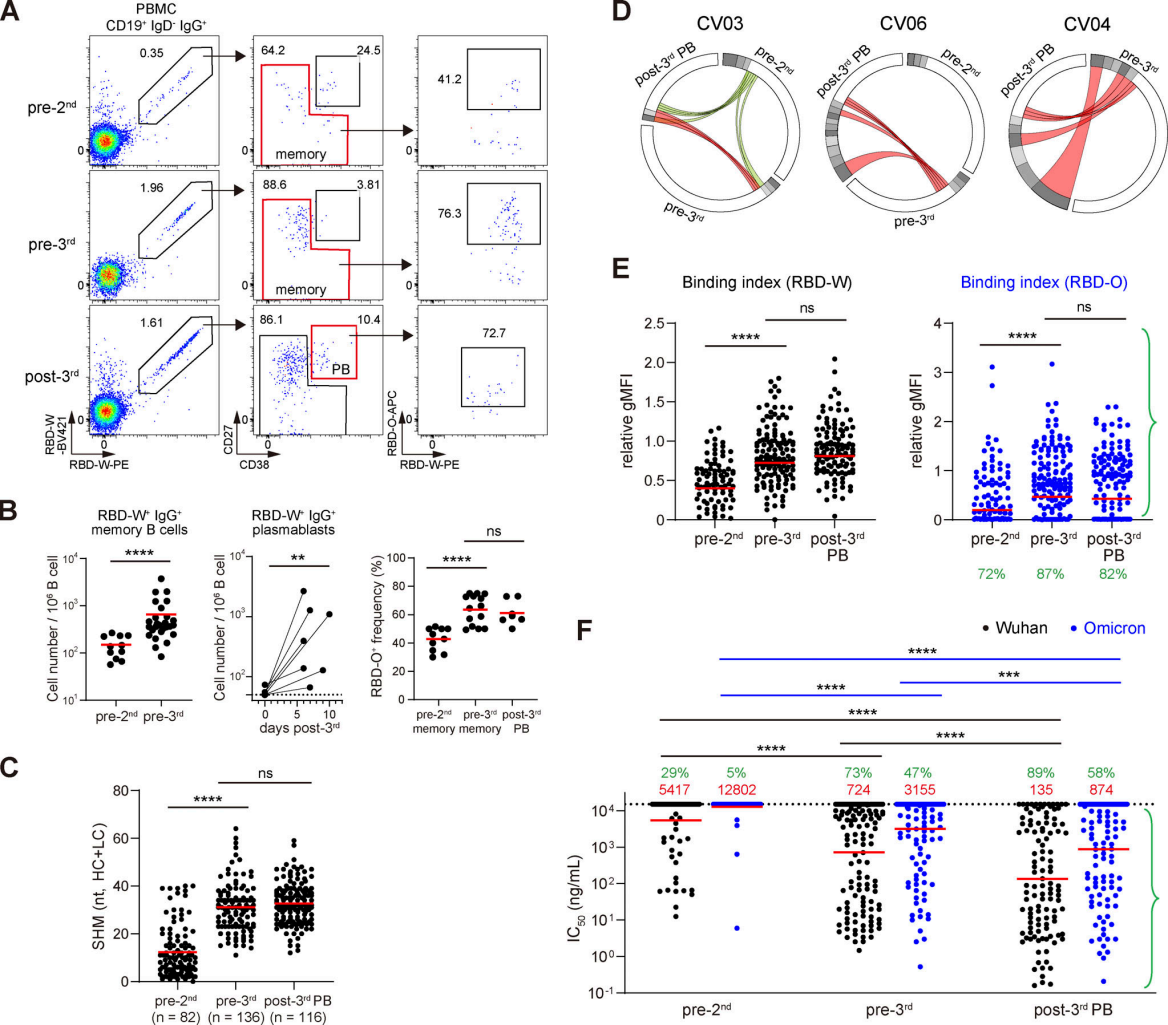
**Analysis of RBD-specific memory B cells, plasmablasts, and the derived antibodies. (A)** Representative flow cytometry plots of RBD-specific IgG^+^ B cells from donors 3 wk after the first (pre-2nd), 9 mo after the second (pre-3rd), and 1 wk after the third (post-3rd) vaccination. The memory B cell and plasmablast populations for single-cell sorting are gated in red. Representative of two independent experiments. **(B)** Left and middle: Total number of RBD-W–specific IgG^+^ memory B cells or plasmablasts in 1 × 10^6^ PBMC B cells. Right: The frequency of RBD-O^+^ cells among RBD-W^+^ IgG^+^ memory B cells and plasmablasts. Red bars represent mean values. **(C)** Number of nucleotide SHM in *IGHV* and *IGLV* in all of the cloned antibodies from the post-3rd plasmablasts. Red bars represent mean values. **(D)** Circos plots showing the BCR clonal relationship between longitudinal pre-2nd memory, pre-3rd memory, and post-3rd plasmablasts. Colored lines indicate the shared clones between different time points, with red lines connecting pre-3rd memory and post-3rd plasmablasts. Gray slices indicate expanded clones, and white slices indicate sequences isolated only once at each time point. **(E)** Bead-based flow cytometric binding assay. Microbeads coated with each monoclonal antibody were tested for binding with PE-labeled RBD-W (left) or RBD-O (right) probes and analyzed by flow cytometry. The binding index was expressed as relative gMFI with the control CR3022 antibody. Red bars represent geometric means. Green percentages indicate the frequency of antibodies with detectable binding. **(F)** Monoclonal antibody neutralization activity against Wuhan and Omicron pseudoviruses expressed as IC_50_ values. Red bars and values represent geometric means. Green percentages indicate the frequency of antibodies with IC_50_ < 15,000 ng/ml. **(E and F)** Experiments were performed in duplicate. **, P < 0.01; ***, P < 0.001; ****, P < 0.0001 by two-tailed Mann–Whitney test.

**Figure S1.**
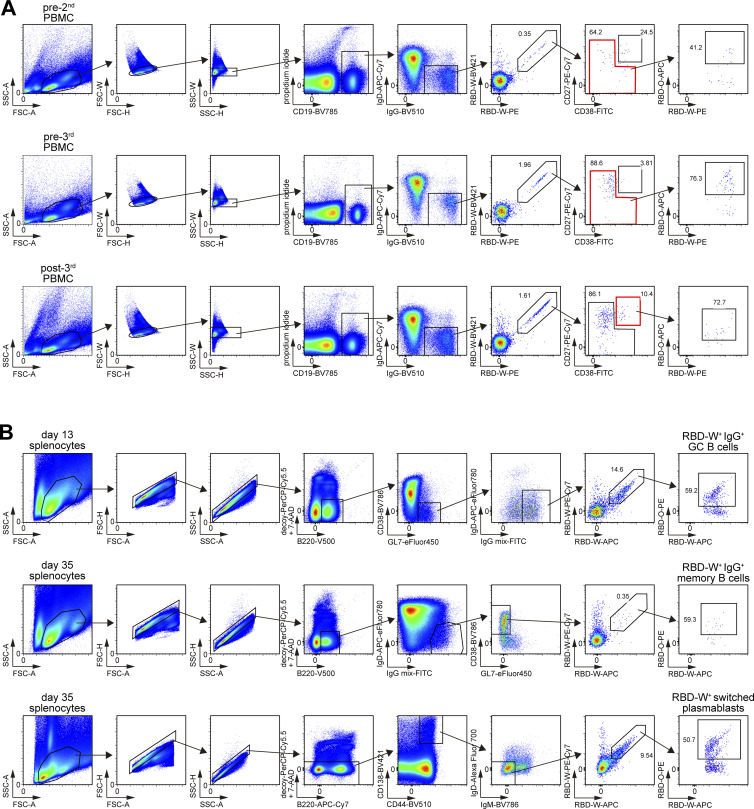
**Gating strategy of flow cytometry analysis. (A)** Gating strategy for human PBMC analysis, related to Fig. 2 A. Data showing representative flow cytometry analysis of pre-2nd, pre-3rd, and 1 wk post-3rd samples. **(B)** Gating strategy of mouse splenocyte analysis, related to Fig. 5. Data showing representative flow cytometry analysis of day 13 GC B cells, day 35 memory B cells, and day 35 (6 d after boost) plasmablasts. Representative of two independent experiments.

A major question is what features of the immune response generated by two-dose vaccination determine the induction of Omicron-reactive antibodies after the third vaccine dose. Given the importance of pre-existing memory B cells for booster-induced antibody responses, we investigated the possibility that the changes in the quality and/or quantity of memory B cells contribute to the differential antibody responses upon the second and third vaccinations. We examined antigen-specific B cells in PBMCs by flow cytometry using fluorescently labeled RBD-W or RBD-O probes. RBD-W–specific IgG^+^ B cells were identified as CD19^+^ IgG^+^ IgD^−^ RBD-W-PE^+^ RBD-W-BV421^+^ and then were sub-fractionated into memory B cells and plasmablasts by CD38/CD27 gating (Fig. 2 A and Fig. S1 A). Individuals who received the second vaccine dose (pre-3rd in Fig. 2 B, left) had higher numbers of IgG^+^ RBD-W–binding memory B cells compared with those who received only one dose (pre-2nd in Fig. 2 B, left). Moreover, the frequency of cells that recognized RBD-O among RBD-W–specific memory B cells significantly increased in the pre-3rd PBMCs compared with that in the pre-2nd samples (Fig. 2 B, right). These results indicate that a second exposure to the original Wuhan vaccine increased the memory B cell fraction, and that these cells are skewed to be more cross-reactive with the Omicron RBD at 9 mo after the second vaccine dose.

To further investigate how RBD-W^+^ RBD-O^−^ and RBD-W^+^ RBD-O^+^ memory B cells were activated by the booster vaccination, we monitored antigen-specific IgG^+^ plasmablasts, demonstrating that the third vaccine dose significantly expanded the IgG^+^ RBD-W^+^ plasmablasts ∼1 wk after vaccination (Fig. 2 B, middle). As shown in Fig. 2 B right, the third vaccine dose gave rise to a similar percentage of RBD-O^+^ plasmablasts among the RBD-W^+^ plasmablasts as that of the pre-3rd memory B cells. Hence, the third dose of vaccination activated a similar proportion of both the pre-3rd Omicron-reactive and Omicron-nonreactive memory B cells, consistent with a previous report (Goel et al., 2022).

### Monoclonal antibody analysis

To examine the quality of memory B cells and plasmablasts, we cloned and characterized 339 monoclonal antibodies from single-cell sorted IgG^+^ RBD-W^+^ memory B cells and plasmablasts from three donors (CV03, CV04, and CV06; Fig. 2 A and Fig. S1 A); CV04 was sampled for memory B cells 9 mo after the second vaccine dose (pre-3rd) and plasmablasts 1 wk after the third vaccine dose (post-3rd); CV03 and CV06 were additionally sampled for memory B cells 3 wk after the first vaccine dose (pre-2nd; Table S1). All of the monoclonal antibodies produced were assessed for binding with RBD-W by a bead-based flow cytometric binding assay (Shinnakasu et al., 2021), and 334 (98.5%) of these antibodies bound to RBD-W, validating the high reliability of our single-cell sorting and antibody cloning experimental pipeline.

**Figure S2.**
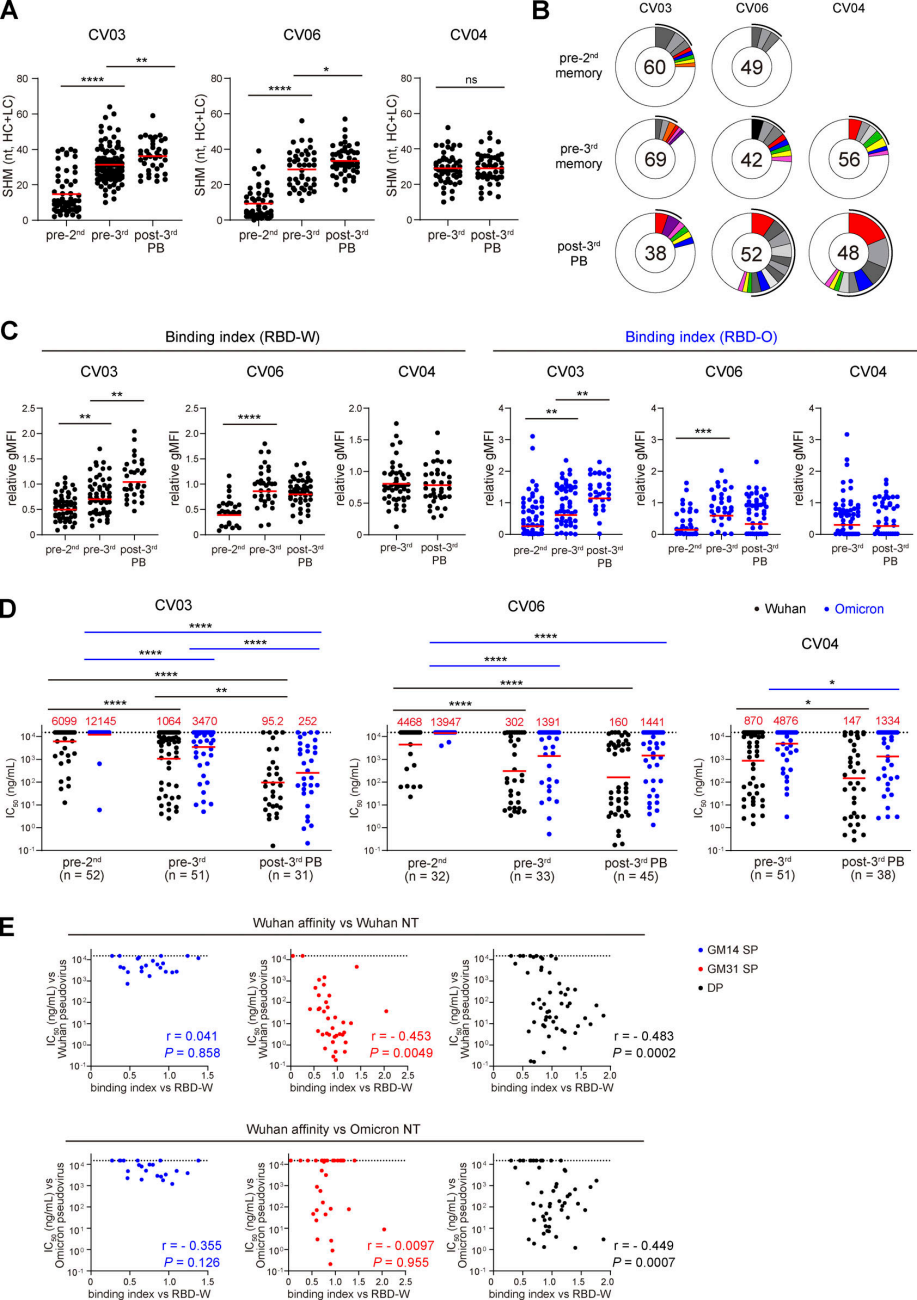
**Monoclonal antibody analysis. (A)** SHM analysis (Fig. 2 C), separated by individual donors. Red bars represent mean values. **(B)** Antibody clonotype analysis showing the distribution of antibody sequences from three donors. The number in the inner circle indicates the number of antibodies cloned. Pie-slice size is proportional to the number of clonally related sequences. Colored slices indicate clonally related antibodies found at multiple time points within the same individual, gray slices indicate expanded clones unique to the time point, and white slices indicate sequences isolated only once per time point. The black outline indicates the frequency of clonally expanded sequences. **(C)** Bead-based flow cytometric binding assay (Fig. 2 E), separated by individual donors. Red bars represent geometric means. **(D)** Pseudovirus neutralization assay (Fig. 2 F), separated by individual donors. Red bars and values represent geometric means. **(C and D)** Experiments were performed in duplicate. **(E)** Pearson correlations showing the correlation coefficient (r) and related significance P value between binding index against RBD-W and IC_50_ against Wuhan pseudovirus (top) or Omicron pseudovirus (bottom), separated into GM14 SP, GM31 SP, and DP clones (Fig. 4 C). *, P < 0.05; **, P < 0.01; ***, P < 0.001; ****, P < 0.0001 by two-tailed Mann–Whitney test (A, C, and D).

SHM analysis of antibody V gene sequences revealed a substantial number of mutations in the post-3rd plasmablasts comparable to that in the pre-3rd memory B cells (Fig. 2 C and Fig. S2 A). Comparison of the antibody repertoires revealed that clonally expanded sequences tended to be more enriched in the post-3rd plasmablasts than in the pre-2nd and pre-3rd memory B cells and that shared clones between the pre-3rd memory B cells and the post-3rd plasmablasts could be detected in all three individuals examined (Fig. 2 D and Fig. S2 B). The affinity of antibodies against RBD antigens was assessed by the bead-based flow cytometric assay. We assigned a relative geometric mean fluorescence intensity (gMFI) binding index to each antibody, which correlates well with the Rmax value of antigen-antibody binding determined by biolayer interferometry (Shinnakasu et al., 2021). Binding indices of antibodies from the pre-3rd memory B cells and the post-3rd plasmablasts against both Wuhan and Omicron RBDs were not significantly different (Fig. 2 E and Fig. S2 C), while antibodies from the post-3rd plasmablasts possessed better neutralizing activity against both Wuhan and Omicron pseudoviruses (Fig. 2 F and Fig. S2 D), which might be due to the enrichment of clonally expanded cells in the post-3rd plasmablasts. Together, these results indicate that the antibody responses after the third dose are preferentially contributed by the pre-3rd memory B cells and not by naive B cells, as was suggested in a previous report (Goel et al., 2022).

We next focused on the quality difference between the pre-2nd and pre-3rd memory B cells. The significantly increased frequency of SHM (Fig. 2 C and Fig. S2 A) and binding indices for RBD-W (Fig. 2 E and Fig. S2 C), as well as functional improvements in neutralization against the Wuhan pseudovirus (Fig. 2 F and Fig. S2 D) of antibodies from the pre-3rd memory B cells compared with those from the pre-2nd memory B cells suggest that the GC reactions induced by the second vaccine dose contribute to the development of more affinity matured and better neutralizing memory B cells. In terms of cross-reactivity of antibodies to the Omicron RBD, the percentage of antibodies with detectable binding indices to RBD-O increased from 72% in pre-2nd to 87% in pre-3rd memory B cells (Fig. 2 E), consistent with the B cell flow cytometry analysis (Fig. 2, A and B), although the percentages of RBD-O^+^ monoclonal antibodies were somewhat higher than those of RBD-O^+^ B cells, probably due to the difference in sensitivity between the two assay systems. The geometric mean of the binding index to RBD-O was significantly increased (Fig. 2 E and Fig. S2 C), and the neutralization potency against the Omicron pseudovirus was significantly enhanced (Fig. 2 F and Fig. S2 D) in the pre-3rd compared with the pre-2nd antibodies, suggesting that the Omicron cross-reactive antibodies also matured during the two dose–induced immune responses, thereby acquiring high neutralization activity.

### Epitopes

**Figure 3.**
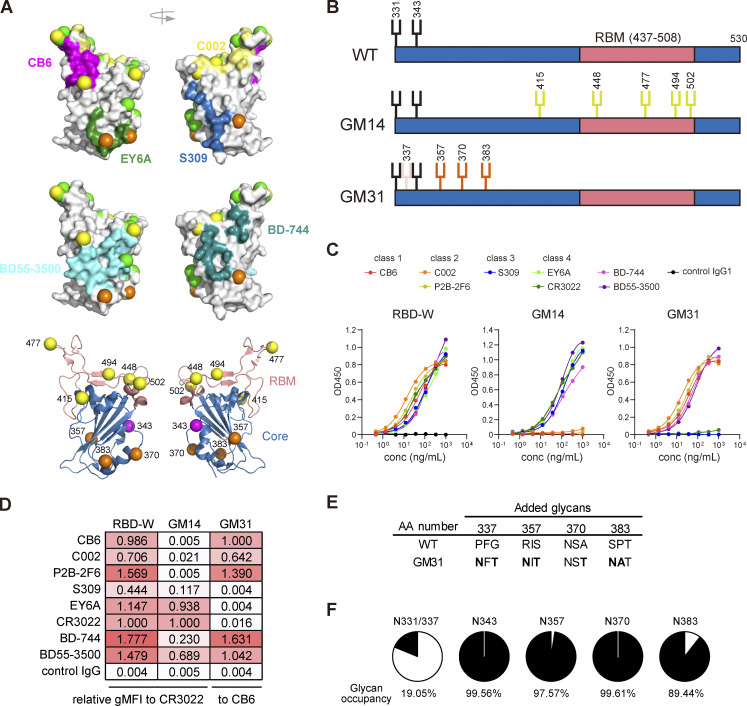
**Development of GM31 probe. (A)** Structural representation showing the antibody epitopes on the RBD (colored surface), Omicron BA.1 mutation (green balls) and GM14 or GM31 glycosylation sites (yellow or orange balls, respectively). **(B)** Schematic illustration of the glycosylation sites in RBD-W WT, GM14, and GM31. The native and additional glycosylation sites are shown in black and colored, respectively. **(C)** Validation of the GM31 probe by an ELISA binding assay using previously reported antibodies. **(D)** Bead-based flow cytometric binding assay using previously reported antibodies. For binding to RBD-W and GM14, gMFI was normalized to CR3022. For binding to GM31, gMFI was normalized to CB6. **(E)** The parental amino acid sequences and introduced NxT sequons. **(F)** Glycan occupancy at N-linked glycosylation sites of GM31 determined by LC/MS. The percentage indicates the glycan occupancy for each site. **(C and D)** Experiments were performed in duplicate.

The increased frequency of RBD-O^+^ cells (Fig. 2 B) and monoclonal antibodies (Fig. 2 E) in the pre-3rd memory B cells compared with the pre-2nd suggested a shift in the distribution of RBD epitope specificity in the memory B cells. To address this possibility, we first attempted to classify antibodies into class 1/2 or class 3/4 epitopes using our N-linked glycan-engineered RBD probes (Duan et al., 2018; Eggink et al., 2014). As previously reported (Shinnakasu et al., 2021), GM14, a Wuhan RBD mutant with five introduced glycosylation sites (NxT motif) in the receptor-binding motif (RBM; Fig. 3, A and B), prevented binding of class 1 (CB6; Shi et al., 2020) and class 2 (C002 [Robbiani et al., 2020] and P2B-2F6 [Ju et al., 2020]) antibodies but maintained epitopes for class 3 (S309; Pinto et al., 2020) and class 4 (EY6A [Zhou et al., 2020] and CR3022 [Yuan et al., 2020]) antibodies (Fig. 3, C and D). Here, we additionally developed a GM31 probe by introducing four NxT sequons into the RBD Core subdomain (Fig. 3, A, B, and E). Liquid chromatography/mass spectrometry (LC/MS) confirmed >89% glycan occupancy at the engineered glycosylation sites (N357, N370 and N383), but ∼19% at N331/N337, in purified GM31 protein expressed in mammalian Expi293 cells (Fig. 3 F). As expected, the introduced glycans on GM31 masked the epitopes for S309, EY6A, and CR3022 antibodies while the binding capacity to CB6, C002, and P2B-2F6 antibodies was intact (Fig. 3, C and D). Consistent with the reported epitopes of BD-744 and BD55-3500 antibodies (Fig. 3 A), which showed potent neutralizing activity toward Wuhan and Omicron BA.1 variants (Cao et al., 2022), these antibodies bound to both GM14 and GM31 (Fig. 3, C and D).

**Figure 4.**
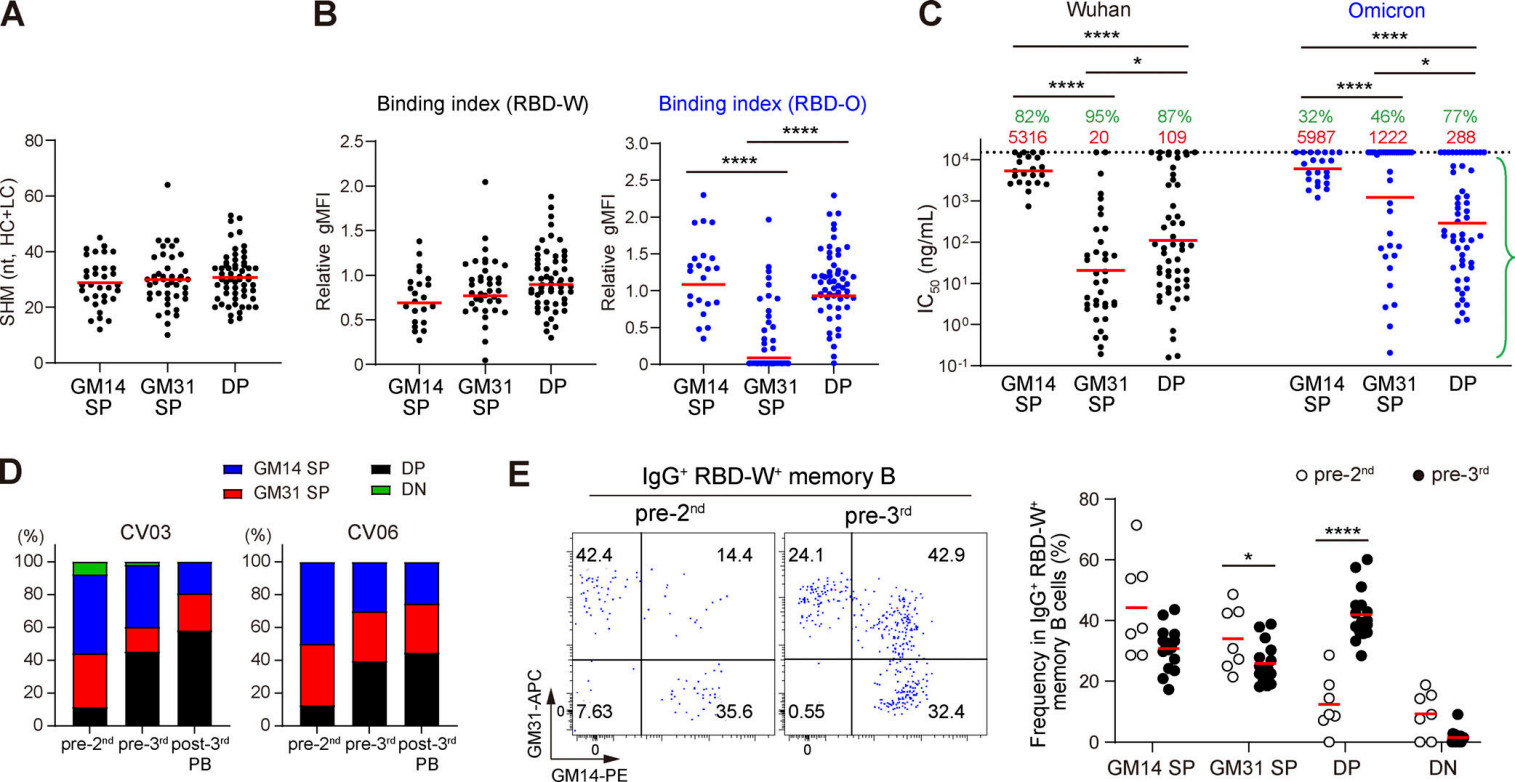
**Epitope shifting analysis using GM14 and GM31 probes. (A)** Number of nucleotide SHM in *IGHV* and *IGLV* in the GM14 SP, GM31 SP, and DP groups of antibodies from the post-3rd plasmablasts. Red bars represent mean values. **(B)** Binding indices of antibodies from the post-3rd plasmablasts measured in Fig. 2 E classified into GM14 SP, GM31 SP, and DP clones. Red bars represent the geometric means. **(C)** Pseudovirus neutralization activity of antibodies from the post-3rd plasmablasts measured in Fig. 2 F classified into GM14 SP, GM31 SP, and DP clones. Red bars and values represent geometric means. Green percentages indicate the frequency of antibodies with IC_50_ < 15,000 ng/ml. **(D)** All the antibodies from CV03 and CV06 donors were tested for GM14 and GM31 binding. Histograms show the frequency of GM14 SP, GM31 SP, DP, and GM14/GM31 double negative (DN) antibodies. **(B–D)** Experiments were performed in duplicate. **(E)** Left: Representative flow cytometry plots of RBD-specific IgG^+^ B cells from the pre-2nd and pre-3rd PBMCs. Right: Frequency of GM14 SP, GM31 SP, DP, and DN cells among RBD-W^+^ IgG^+^ memory B cells. Representative of two independent experiments. *, P < 0.05; ****, P < 0.0001 by two-tailed Mann–Whitney test.

Using the bead-based flow cytometry assay, we first tested all the antibodies from the post-3rd plasmablasts for binding with fluorescently labeled GM14 and GM31 probes. We found that the antibodies were classified into GM14^+^ GM31^−^ (GM14 single positive [SP]; *n* = 22 [19%]), GM14^−^ GM31^+^ (GM31 SP; *n* = 38 [33%]), or GM14^+^ GM31^+^ (GM14/GM31 double positive [DP]; *n* = 56 [48%]) groups. To compare the functionality between GM14 SP, GM31 SP, and DP antibodies, SHM, binding index, and neutralization activity data (Fig. 2, C, E, and F) were categorized into these three groups. While SHM numbers and the affinity to RBD-W were comparable between the groups (Fig. 4, A and B), GM31 SP antibodies had a significantly lower binding index to RBD-O than GM14 SP and DP antibodies (Fig. 4 B), as expected due to the positions of the mutated RBM residues in the Omicron BA.1 variant (Fig. 3 A). Neutralization activity among the three groups was the lowest in GM14 SP antibodies against both Wuhan and Omicron pseudoviruses (Fig. 4 C), also consistent with the previous data showing that the antibodies targeting the RBD Core are less potently neutralizing (Starr et al., 2021). DP antibodies showed a slightly lower neutralizing potency against Wuhan pseudovirus compared with GM31 SP antibodies. In contrast, DP antibodies possessed a higher frequency of (77 versus 46%) and more potent (288 versus 1,222 ng/ml IC_50_) Omicron-neutralizing activities than the GM31 SP antibodies (Fig. 4 C). The DP antibodies showed a significant correlation between the binding index to RBD-W and neutralization potency against Omicron pseudovirus in contrast to GM14 SP and GM31 SP antibodies (Fig. S2 E), suggesting that they target to the conserved RBD region. Taken together, both GM14 SP and DP antibodies could bind to RBD-O, but the latter had more potent Omicron-neutralizing activity.

We then tested all the antibodies from the pre-2nd and the pre-3rd memory B cells of CV03 and CV06 donors for binding to GM14 and GM31 probes. We found that the majority of antibodies from the pre-2nd memory B cells were classified into GM14 SP or GM31 SP and that the frequency of the DP antibodies substantially increased in the pre-3rd memory B cells and the post-3rd plasmablasts in both donors (Fig. 4 D). Furthermore, by flow cytometry analyses of multiple PBMC samples, we confirmed that the frequency of the DP cells among IgG^+^ RBD-W^+^ memory B cells significantly increased in the pre-3rd compared to the pre-2nd (Fig. 4 E), suggesting the shift in the distribution of RBD-W–specific memory B cell epitopes after the second vaccine dose.

**Figure S3.**
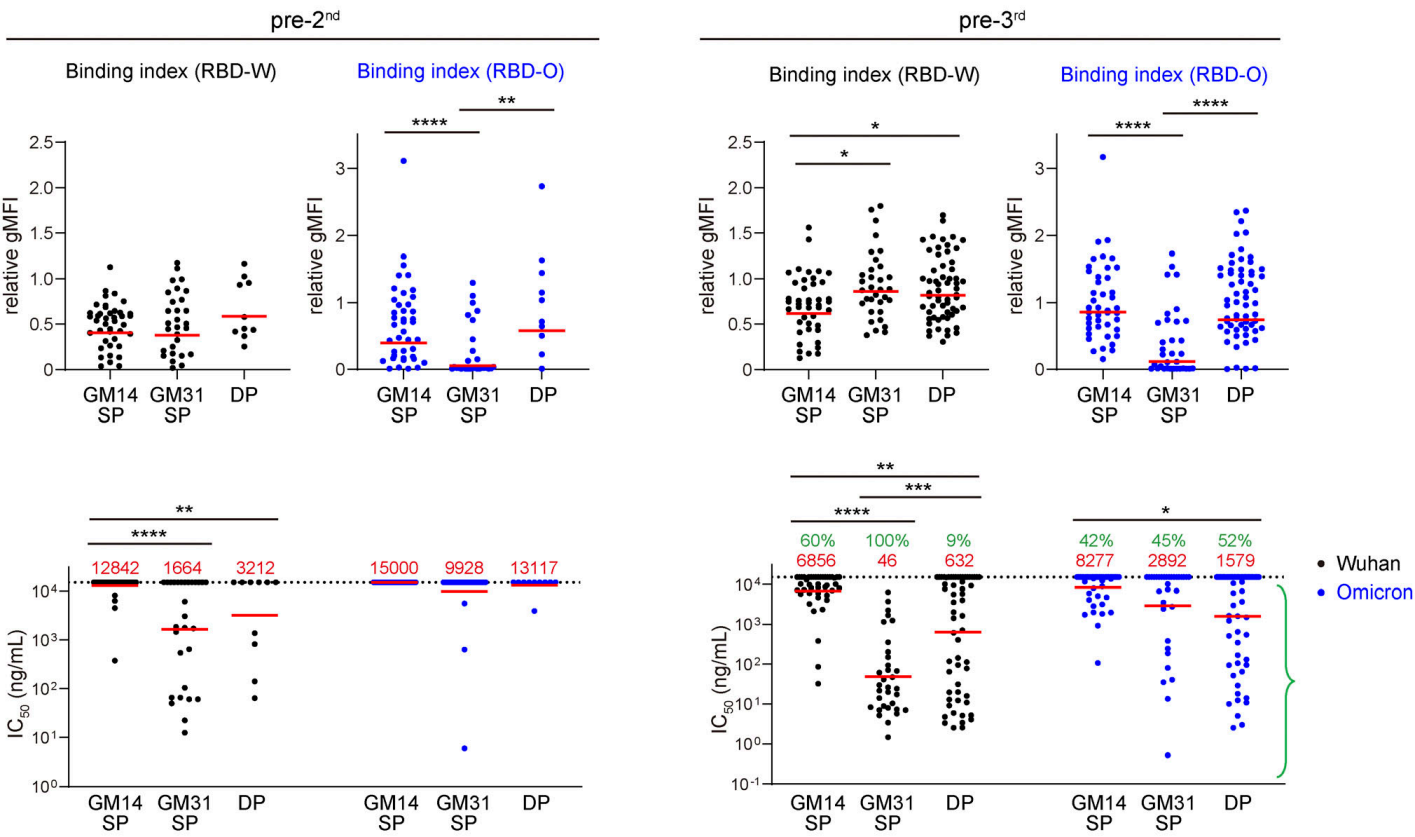
**Binding indices and pseudovirus neutralization activity of antibodies from the pre-2nd and pre-3rd memory B cells.** Related to Fig. 4, B and C. Bead-based cytometric binding assay and pseudovirus neutralization activity of antibodies from the pre-2nd and pre-3rd memory B cells measured in Fig. 2, E and F were classified into GM14 SP, GM31 SP, or DP clones. Red bars and values represent geometric means. Green percentages indicate the frequency of antibodies with IC_50_ < 15,000 ng/ml. Experiments were performed in duplicate. *, P < 0.05; **, P < 0.01; ***, P < 0.001; ****, P < 0.0001 by two-tailed Mann–Whitney test.

Hence, in regard to the two dose–induced antibody responses, promptly produced antibodies are likely to be generated by GM14 SP and GM31 SP memory B cells at pre-2nd (Fig. 4, D and E). As seen in Fig. S3 at pre-2nd, these memory B cells manifested barely neutralization activities toward Omicron pseudovirus, explaining for only a limited neutralizing antibody response toward Omicron variant after the second dose (Fig. 1 C).

### Validation of the concept

Given that the post-2nd serum antibody reacts with RBD-W, but barely cross-reacts with RBD-O (Fig. 1 B), we hypothesized that the antibodies generated before and/or after the second vaccine dose masked the immunodominant epitopes on RBD-W, which modulated the GC and memory B cell repertoires and eventually generating more Omicron-reactive memory B cells. For validation of this antibody feedback concept, we conducted two sets of experiment using our previously established mouse immunization model (Shinnakasu et al., 2021).

**Figure 5.**
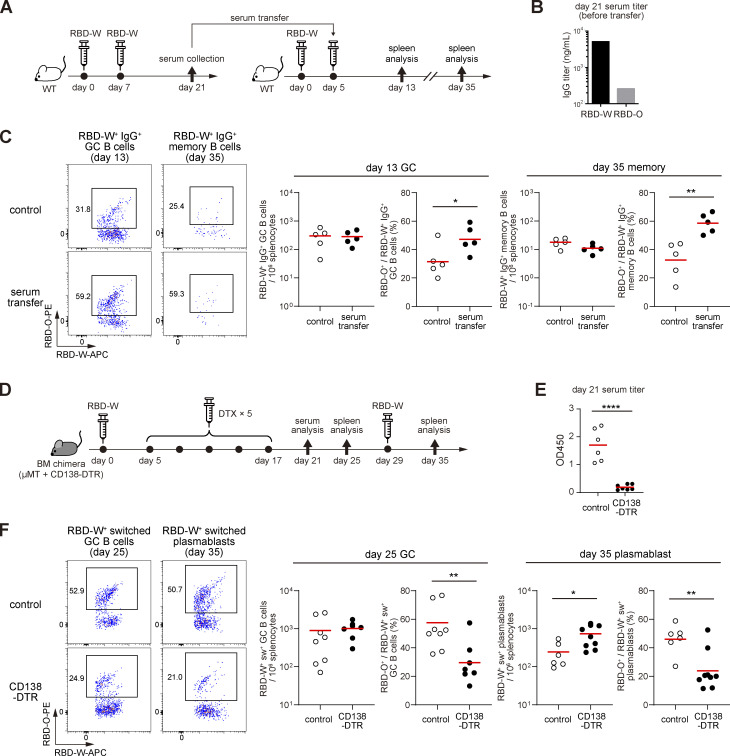
**Proof-of-concept experiment of antibody feedback with mouse immunization model. (A)** Schematic of experimental design for B and C. **(B)** Serum anti–RBD-W and anti–RBD-O IgG titers collected 21 d after immunization of wild-type mice with RBD-W. **(C)** Left: Flow cytometry analysis of day 13 or day 35 splenocytes of mice immunized with RBD-W on day 0 and injected with serum on day 5. Right: Total cell number of RBD-W^+^ IgG^+^ GC or memory B cells and summary of the frequency of RBD-O^+^ cells among RBD-W^+^ IgG^+^ GC or memory B cells. **(D)** Schematic of the experimental design for E and F. **(E)** Serum anti–RBD-W IgG titers collected on day 21 after immunization and DTX administration from BM chimeras reconstituted with mixed BM cells from μMT (80%) and CD138-DTR (20%) mice. Control groups were reconstituted with μMT (80%) and wild-type (20%) BM cells. **(F)** Left: Flow cytometry analysis of day 25 or day 35 splenocytes of BM chimeras. Right: Total cell number of RBD-W^+^ switched GC B cells or plasmablasts and summary of the frequency of RBD-O^+^ cells among RBD-W^+^–switched GC B cells or plasmablasts. Red bars represent means. Representative of two independent experiments. *, P < 0.05; **, P < 0.01; ****, P < 0.0001 by unpaired two-tailed Student’s *t* test.

First, wild-type C57BL/6 mice were immunized with RBD-W conjugated to streptavidin-coated polystyrene microspheres together with AddaVax adjuvant, boosted on day 7 and then serum was collected on day 21 (Fig. 5 A). The serum showed ∼20-fold higher IgG titer against RBD-W than RBD-O (Fig. 5 B). Another wild-type mouse was immunized with RBD-W antigen and then injected with the collected serum on day 5. 8 or 30 d after the serum transfer, we found a significantly increased frequency of RBD-O^+^ cells among RBD-W^+^ IgG^+^ GC or memory B cells than in controls, respectively, while the total RBD^+^ GC and memory B-cell numbers were not significantly altered (Fig. 5 C and Fig. S1 B).

Second, as a complementary approach, we attempted to decrease the induced antibody levels during the immune response using CD138-DTR mice (Fig. 5 D; Vijay et al., 2020). Mixed bone marrow (BM) chimeras reconstituted with BM cells from μMT and CD138-DTR mice were immunized with RBD-W, the same as in Fig. 5 A, then diphtheria toxin (DTX) was administered five times from day 5 to day 17 to deplete CD138^+^ B cell lineage cells composed of plasmablasts and plasma cells (Vijay et al., 2020). As expected, the serum anti–RBD-W IgG titer on day 21 in CD138-DTR mice was much lower than controls (Fig. 5 E). On day 25, the frequency of RBD-O^+^ cells among RBD-W^+^ switched GC B cells was significantly decreased compared to control mice (Fig. 5 F). Due to the technical difficulty in reliably detecting rare memory B cells in this experimental setting, we instead evaluated the memory recall response by analyzing the antigen-specific plasmablasts 6 d after boosting these mice with the same RBD-W antigen on day 29. The frequency of RBD-O^+^ cells among RBD-W^+^ switched plasmablasts was significantly decreased compared to controls (Fig. 5 F and Fig. S1 B). While total RBD^+^ GC B cell numbers were not altered, the total number of RBD-W^+^ plasmablasts was increased in CD138-DTR mice, possibly due to the activation of even low-affinity memory B cells in the context of the decreased levels of pre-existing anti-RBD antibodies. These results demonstrated that the addition or depletion of Omicron-nonreactive antibodies led to the increased or decreased frequency of Omicron-reactive GC B cells and subsequent memory B cells, respectively. Although low titer, low affinity, or multi-epitope antibodies are known to enhance humoral responses by creating immune complexes (Heesters et al., 2014), overall RBD-specific GC numbers were not significantly affected by these interventions (Fig. 5, C and F), suggesting that epitope masking predominantly contributes to the observed modulations in the GC and memory B cell repertoires in our experimental settings.

## Discussion

Based on animal models using passive transfer of polyclonal serum or monoclonal antibodies, it has been known for decades that antibody feedback regulates the humoral immune response (Grantham and Fitch, 1975; Henry and Jerne, 1968; Heyman, 2000; Karlsson et al., 1999; Zhang et al., 2013). More recent studies using malaria, HIV, or SARS-CoV-2 antigen-specific transgenic B cell receptor (BCR) knock-in mouse models have demonstrated that high titers of epitope-specific and high-affinity antibodies limit activation of the cognate naïve and memory B cells likely through epitope masking (McNamara et al., 2020; Tas et al., 2022). Furthermore, in the context of human vaccination, introduction of epitope-specific monoclonal antibodies is reported to bias memory B cell selection (Schaefer-Babajew et al., 2022).

Here, our data suggest that the antibodies induced during immune responses shift the distribution of epitopes recognized by memory B cells away from the immunodominant RBD epitope, which facilitates the accumulation of Omicron-reactive memory B cells acquiring high neutralization potency during ∼9 mo after two doses of the mRNA vaccine. We have also proven this concept using immunized mouse models in non-transgenic settings. Although the generated memory B cells barely contribute to circulating antibodies before the third vaccine dose, upon homologous antigen rechallenge, they promptly produce large amounts of neutralizing antibodies. Since the titer and the affinity of anti-RBD antibodies prior to the second vaccine dose are low, we rather prefer the idea that antibodies generated by boosted memory B cells play a major role in this feedback mechanism presumably due to epitope masking. Supporting this idea, by the adoptive transfer experiments in mouse models, antibodies produced by IgG^+^ memory B cells were shown to be able to inhibit the formation of secondary GCs by IgM^+^ memory B cells (Pape et al., 2011).

Given the recent evidence that the frequency of Omicron-cross-reactive memory B cells is increased at a late time point (5 mo after two doses) compared with 1 mo after (Kotaki et al., 2022), these IgG^+^ memory B cells are likely to be constantly generated for a relatively long period. Considering that the almost threefold increase in the numbers of somatic mutations 9 mo after the second dose immunization in our study, together with the recent findings that the two-dose mRNA vaccine induces a robust GC B cell response lasting for at least 6 mo after vaccination (Kim et al., 2022; Mudd et al., 2022; Turner et al., 2021), many of the IgG^+^ memory B cells are probably generated through GC responses. Such further SHM contributes to affinity maturation as well as potentiating neutralizing activity on Omicron-reactive memory B cells. According to the above scenario, in the context of post-vaccination infection, an efficient development of the breadth observed after the continuous vaccination may not occur. Assuming that sufficient neutralizing antibodies are generated after vaccination, the infecting viruses might be simply cleared by preventing their spread and cleaning their antigens, thereby halting both GC reactions and epitope-specific masking.

Previous adoptive transfer experiments of memory B cells into intact recipient mice showed that, in the case of homologous booster immunization, IgG^+^ and IgM^+^ B cells possess more intrinsic capability to be differentiated into plasma cells and GCs, respectively (Inoue et al., 2021b; Quast and Tarlinton, 2021; Weisel and Shlomchik, 2017). Because we focused on the IgG^+^ memory B cells in vaccinated individuals in this study, our data cannot exclude the following two possibilities. First, IgM^+^ memory B cells or naive B cells enter the GC after the second vaccine dose, and subsequently differentiate to affinity-matured IgG^+^ memory B cells. Alternatively, already generated IgG^+^ memory B cells re-enter the GC, albeit less efficiently, after the second vaccine dose.

In conclusion, masking of immunodominant epitope by antibodies diversifies immunogenicity of otherwise subdominant epitopes. In the case of SARS-CoV-2 mRNA vaccination, these second layer antibodies target conserved RBD epitopes and acquire high potency neutralizing activity by SHM, contributing to the development of functional breadth.

## Materials and methods

### Human subjects and sampling

Human blood samples were collected at Hakodate National Hospital, Hokkaido University, and Osaka University, and plasma and PBMCs were isolated using Leucosep Tube (Greiner Bio-One) or Lymphoprep Tube (Serumwerk Bernburg) according to the manufacturer’s instructions. The study protocol was approved by the institutional review board of Hakodate National Hospital (permit no. R4-0912002), Hokkaido University Hospital (permit no. 021–0157), Osaka University Hospital (permit no. 907), and Osaka University (permit no. IFReC-2021-4-2, 898-5). All volunteers provided written informed consent in accordance with the Declaration of Helsinki.

### Mice

CD138-DTR (Vijay et al., 2020) and μMT mice (Kitamura et al., 1991) were described previously. C57BL/6 mice were purchased from CLEA Japan and SLC Japan. For mixed BM chimera production, C57BL/6 mice were lethally irradiated by x ray (8.5 Gy) and 6 h later, the mice were injected intravenously with mixed BM cells from μMT (80%) and CD138-DTR (20%) mice. Control groups were reconstituted with 80% μMT and 20% wild-type BM cells. Chimeric mice were rested for at least 8 wk before immunization. Mice were bred and maintained under specific pathogen–free conditions and all animal experiments were performed under the institutional guidelines of Osaka University.

### Recombinant protein expression and purification

The mammalian expression constructs for SARS-CoV-2 Wuhan-Hu-1 spike RBD and GM14 were described previously (Shinnakasu et al., 2021). Briefly, RBDs were conjugated with N-terminal signal peptide and C-terminal 6×His-Avi-tag. The expression vector of the Omicron BA.1 spike RBD contains the following mutations; G339D/S371L/S373P/S375F/K417N/N440K/G446S/S477N/T478K/E484A/Q493R/G496S/Q498R/N501Y/Y505H. For GM31 construction, NxT sequons were introduced in the Core domain of RBD-W (Fig. 3, A, B, and E) so that the structure of RBD was not disrupted by the mutations with their glycosylation score >0.5 by NetNGlyc (https://services.healthtech.dtu.dk/service.php?NetNGlyc-1.0). Recombinant proteins were expressed and purified using the Expi293 Expression System (Thermo Fisher Scientific) and TALON metal affinity resin (Clontech) as described (Inoue et al., 2021a). Biotinylation of RBDs was performed by cotransfection of a BirA enzyme expression plasmid (#64395; Addgene) and expression in culture with 100 μM biotin (Sigma-Aldrich). Biotinylation efficiency was confirmed as >90% by SDS-PAGE.

### N-linked glycan occupancy analysis by LC/MS

Sample preparation and LC/MS analysis were performed as previously described (Shinnakasu et al., 2021).

### Immunization

Mice were immunized by intraperitoneal injection with 30 μg biotinylated RBD-W preincubated with 7.5 μg streptavidin-coated 0.1-μm microspheres (Bangs Laboratories) and 37.5 μl AddaVax adjuvant (InvivoGen) as described (Shinnakasu et al., 2021). For serum transfer experiments, serum that had been collected and pooled from 10 immunized mice was injected intraperitoneally (130 μl/mouse). For DTX administration, mice were injected intraperitoneally with 500 ng DTX (Sigma-Aldrich) in PBS once every 3 d for a total of 5 times.

### Flow cytometry and cell sorting

Single-cell suspensions of human PBMCs and mouse splenocytes were analyzed and sorted on FACSCanto II (BD), Attune NxT (Thermo Fisher Scientific) or FACSAria II (BD). For human PBMC staining, cells were thawed at 37°C and immediately washed with PBS containing 2% FBS, followed by staining with RBD probes for 30 min at room temperature. Cells were then washed and stained with antibodies in Brilliant Stain Buffer Plus (BD) for 30 min at room temperature. For mouse splenocytes, cells were prestained with a decoy probe to gate out cells that non-specifically bound streptavidin, and then stained with RBD probes and antibodies in PBS with 2% FBS for 20 min at 4°C. BV510 anti-human IgG, FITC anti-human CD38, V500 anti-mouse B220, BV786 anti-mouse IgM, FITC anti-mouse IgG1 (for IgG mix), FITC anti-mouse IgG2a (for IgG mix), FITC anti-mouse IgG3 (for IgG mix), BV786 anti-mouse CD38, PE Streptavidin, BV421 Streptavidin, and PE-Cy7 Streptavidin were purchased from BD. BV785 anti-human CD19, APC-Cy7 anti-human IgD, BV510 anti-mouse/human CD44, APC-Cy7 anti-mouse/human B220, BV421 anti-mouse CD138, Alexa Fluor 700 anti-mouse IgD, FITC anti-mouse IgG2b (for IgG mix), and 7-AAD (as viability dye) were purchased from BioLegend. PE-Cy7 anti-human CD27, eFluor450 GL-7, APC Streptavidin, and PerCP-Cy5.5 Streptavidin were purchased from Thermo Fisher Scientific. Propidium iodide (as a viability dye) was purchased from Sigma. RBD-W, RBD-O, GM14, and GM31-specific cells were detected by biotinylated RBDs prelabeled with fluorophore-conjugated streptavidin. Data were analyzed using FlowJo software v10.8 (BD).

### BCR cloning and antibody expression

RBD-W–specific IgG^+^ memory B cells (CD19^+^ IgG^+^ IgD^−^ RBD-W-PE^+^ RBD-W-BV421^+^ CD27^lo/int^ CD38^lo/int^) and plasmablasts (CD19^+^ IgG^+^ IgD^−^ RBD-W-PE^+^ RBD-W-BV421^+^ CD27^hi^ CD38^hi^) in human PBMCs were single-cell sorted into 96-well plates containing 4 μl/well of ice-cold 0.5× PBS with 10 mM dithiothreitol, 1.6 U RNasin Plus RNase Inhibitor (Promega) and 0.1 U SUPERase-In RNase Inhibitor (Thermo Fisher Scientific). cDNA was synthesized in a total volume of 10 μl/well in the original 96-well plates containing 100 ng random primer (pd(N)6; Sigma-Aldrich), 0.5 μl of 10 mM each dNTPs (QIAGEN), 0.33% (v/v) NP-40, 5 U SuperScript IV reverse transcriptase, and 1× RT buffer (Thermo Fisher Scientific) by incubating at 23°C for 10 min, 50°C for 10 min and then 80°C for 10 min. BCR cloning and monoclonal antibody expression were performed as described previously (Inoue et al., 2021a; Tiller et al., 2008) with the following modifications. PCR-amplified Igγ and Igκ or λ V(D)J transcripts were cloned into the human Igγ1/Igκ (pVITRO1-dV-IgG1/κ, #52213; Addgene) or Igγ1/Igλ-expression vector (pVITRO1-dV-IgG1/λ, #52214; Addgene; Dodev et al., 2014), respectively, using the SLiCE method (Motohashi, 2015). Monoclonal antibodies were expressed using the Expi293 Expression System (Thermo Fisher Scientific) and purified from the culture supernatants of Expi293F cells by Protein G Mag Sepharose (Cytiva).

### BCR clonotype definition

For each sequence, fasta files were made and then annotated using igblastn in AIRR format using Change-O against the IMGT reference database (Gupta et al., 2015). Contigs of heavy-chain BCRs were grouped into clonotypes with the DefineClones.py script using the following sequential criteria: (1) at least 80% amino acid sequence similarity at the junction of the CDR3 based on hamming distance; (2) the junction of the CDR3 was normalized by the number of amino acids sequence.

### ELISA

The 96-well plates (Nunc MaxiSorp, Thermo Fisher Scientific) were coated with 2 μg/ml of RBD-W or RBD-O for the capture of antibodies. After blocking with BlockingOne reagent (Nacalai), the plates were incubated with serially diluted plasma or monoclonal antibodies. RBD-specific IgG antibodies were detected using horseradish peroxidase–conjugated goat anti-human IgG (Southern Biotech) with SureBlue TMB substrate (KPL). The absorbance at 450 nm was measured with a microplate reader (ARVO X3, PerkinElmer). EY6A control antibody was included on each plate for plasma samples to convert OD values into relative antibody concentrations.

### Bead-based flow cytometric binding assay

OneComp eBeads compensation beads (Thermo Fisher Scientific) were incubated with mouse anti-human IgG (BD), followed by coating with 600 ng monoclonal antibodies (hIgG1/hIgκ or hIgλ) to be tested. After washing with PBS containing 2% FBS, particles were incubated with PE-conjugated RBD probes for 20 min at room temperature. Binding capacity of antibodies to each probe was assessed by flow cytometry (FACSCanto II [BD] or Attune NxT [Thermo Fisher Scientific]) and quantified as relative gMFI values with control antibodies. CR3022 was used as control for RBD-W, RBD-O, and GM14, and CB6 was used as control for GM31. For the binding assay with the GM14 and GM31 probes, antibodies with a relative gMFI value of >0.1 against both probes were defined as a GM14/GM31 DP clones.

### Pseudovirus neutralization assay

Preparation of SARS-CoV-2 Wuhan-Hu-1 and Omicron BA.1 spike protein-pseudotyped VSVΔG-luc has been described previously (Shinnakasu et al., 2021; Tani et al., 2010; Yoshida et al., 2021). Briefly, HEK293T cells were transfected with expression plasmids for Wuhan-Hu-1 and Omicron BA.1 spike protein using TransIT-LT1 Transfection Reagent (Mirus) according to the manufacturer’s instructions. After 24 h, cells were infected with VSVΔG-luc virus for 2 h and then washed with DMEM and further incubated for 24 h. Cell-free supernatant was harvested and used for the neutralization assay as described previously (Nie et al., 2020). Human plasma samples were inactivated at 56°C for 30 min prior to the neutralization assay. Spike-pseudotyped VSVΔG-luc was incubated with serial dilutions of human plasma or recombinant antibodies for 1 h at 4°C, and then inoculated onto a monolayer culture of VeroE6-TMRPSS2 cells (JCRB1819; NIBION) in a 96-well plate. After 24 h, luciferase activity was measured by Luciferase Assay System (Promega) and GloMax Discover luminometer (Promega). The NT_50_ values for plasma or the half-maximal inhibitory concentration (IC_50_) values for monoclonal antibodies were determined as described (Nie et al., 2020).

### Statistical analysis

Statistical analysis was performed using Prism 8 (Graphpad). Paired data were analyzed with two-tailed Wilcoxon test, and unpaired data were analyzed with two-tailed Mann–Whitney test or Student’s *t*-test.

### Online supplemental material

Table S1 summarizes the human sample information. Fig. S1 shows the full gating strategy for human PBMCs (related to Fig. 2 A) and mouse splenocytes (related to Fig. 5). Fig. S2 shows monoclonal antibody analysis, separated by individual donors (related to Fig. 2). Fig. S3 shows binding indices and neutralization activity of monoclonal antibodies from the pre-2nd and pre-3rd memory B cells (related to Fig. 4, B and C).

## Supplementary Material

Table S1 summarizes the human sample information.
